# Sex-specific differences in vascular circulation and metabolic regulation associated with aging and cardiovascular health problems

**DOI:** 10.3389/fragi.2026.1822261

**Published:** 2026-05-28

**Authors:** Andrzej Marcinek, Joanna Katarzynska, Jerzy Gebicki

**Affiliations:** 1 Institute of Applied Radiation Chemistry, Lodz University of Technology, Lodz, Poland; 2 Angionica Ltd., Lodz, Poland

**Keywords:** aging, flow mediated skin fluorescence, metabolic (mitochondrial) regulation, NADH/NAD^+^ redox balance, sex-specific differences, vascular circulation

## Abstract

The Flow Mediated Skin Fluorescence–Post-Occlusive Reactive Hyperemia (FMSF–PORH) technique proves that the skin can serve as an easily accessible and sensitive model for monitoring systemic redox changes in the NADH/NAD^+^ balance. The three most important diagnostic parameters of the method are related to the response to transient ischemia. Hyperemic Response (HR_max_) characterizes the hyperemia phase and reflects the rapid increase in macrocirculatory blood flow after occlusion of the brachial artery. Hypoxia Sensitivity (HS) characterizes the reperfusion phase, by assessing the activation of myogenic microcirculatory oscillations. Ischemic Response (IR_max_) is related to the ischemia phase and reflects the adaptation of cellular metabolism to ischemia. All three FMSF–PORH parameters depend on health condition and age. Sex-specific differences in the FMSF–PORH parameters are not seen in young healthy individuals, but appear with cardiovascular health problems and/or advanced age. Females compared to males have a significantly lower the IR_max_ parameter, characterizing metabolic (mitochondrial) regulation. In contrast, the HR_max_ parameter, characterizing macrocirculation, is higher in females than in males. All parameters slowly deteriorate with age and cardiovascular health problems. The results of this study highlight the need for a sex-specific perspective on vascular and metabolic health, as well as differentiated preventive healthcare strategies.

## 1 Introduction

Metabolic disorders related to obesity, insulin resistance and diabetes can negatively affect vascular health. Systemic inflammation and oxidative stress are major contributors to cardio-metabolic diseases, but their precise regulatory mechanisms and impact on vascular redox signalling are not fully understood. A key role is played by the NADH/NAD^+^ redox balance. Imbalances (too much NADH or too little NAD^+^) disrupt energy production, antioxidant defence and signalling pathways, signalling disease (Xiao and Loscalzo, 2020; Mercola, 2025; Yang et al., 2025). The pathological relevance of the NADH/NAD^+^ redox imbalance is particularly promising for diagnosing cardiovascular disease and diabetes (Wu et al., 2016; Yan, 2021). However, there are few diagnostic approaches based on assessing the NADH/NAD^+^ redox balance and applying this concept in medical diagnostics remains challenging.

Recently, a new diagnostic technique named Flow Mediated Skin Fluorescence–Post-Occlusive Reactive Hyperemia (FMSF–PORH) has been developed, aimed at non-invasive assessment of vascular circulation and metabolic regulation (Katarzynska et al., 2019). This technique is based on vascular and metabolic responses to transient ischemia, measured by changes in skin NADH fluorescence. Numerous studies have documented the diagnostic potential of the FMSF–PORH technique for analysing various diseases and disorders (Marcinek et al., 2023; Marcinek et al., 2024; Marcinek et al., 2025b; Marcinek et al., 2025a; Mikosiński et al., 2023). Using this technique, skin can serve as an easily accessible and sensitive model for monitoring systemic redox changes.

The very high sensitivity of the FMSF-PORH technique allows for precise analysis of the NADH/NAD^+^ redox balance. Recording NADH fluorescence across various phases of the measurement allows for quantitative assessment of both macro- and microcirculation function (hyperemic and reperfusion phases), as well as mitochondrial function (ischemic phase). Diagnostic use of the FMSF–PORH technique in tests involving hundreds of individuals, both healthy and sick, prompted us to quantify the parameters responsible for vascular and metabolic (mitochondrial) function, enabling vascular and metabolic health status to be monitored separately.

It is generally known that vascular circulation and metabolic regulation are quite different in females compared to males. However, our understanding of these differences is quite limited, particularly in terms of microcirculatory and mitochondrial functions (Patel et al., 2005; Skaug et al., 2013; Blum, 2023; Fekete et al., 2025; Freedman et al., 2026). There is also limited knowledge concerning the effects of age on these functions in females compared to males.

In this study, we used the FMSF–PORH technique to investigate sex-specific differences in vascular circulation and metabolic regulation in relation to the age of patients with cardiovascular health problems. The FMSF–PORH technique is the only non-invasive technique that allows for the simultaneous assessment of both vascular circulation and metabolic regulation in a single measurement. The observed differences are pronounced and highlight the need for a sex-specific perspective on vascular and metabolic health, as well as differentiated preventive healthcare strategies.

## 2 Methods

### 2.1 The FMSF–PORH methodology

The Flow Mediated Skin Fluorescence (FMSF) method measures nicotinamide adenine dinucleotide (NADH) fluorescence from skin tissue cells. Skin fluorescence is an extremely sensitive sensor of changes in blood circulation, the delivery of oxygen and other nutrients to the epidermal cells and the mitochondrial response to those changes. When combined with brachial artery occlusion for 3 min, this technique is called Flow Mediated Skin Fluorescence–Post-Occlusive Reactive Hyperemia (FMSF–PORH). The FMSF–PORH technique has been described in detail in numerous publications (Katarzynska et al., 2019; Marcinek et al., 2023; Marcinek et al., 2025a) and will not be discussed in more detail here.

**FIGURE 1:**
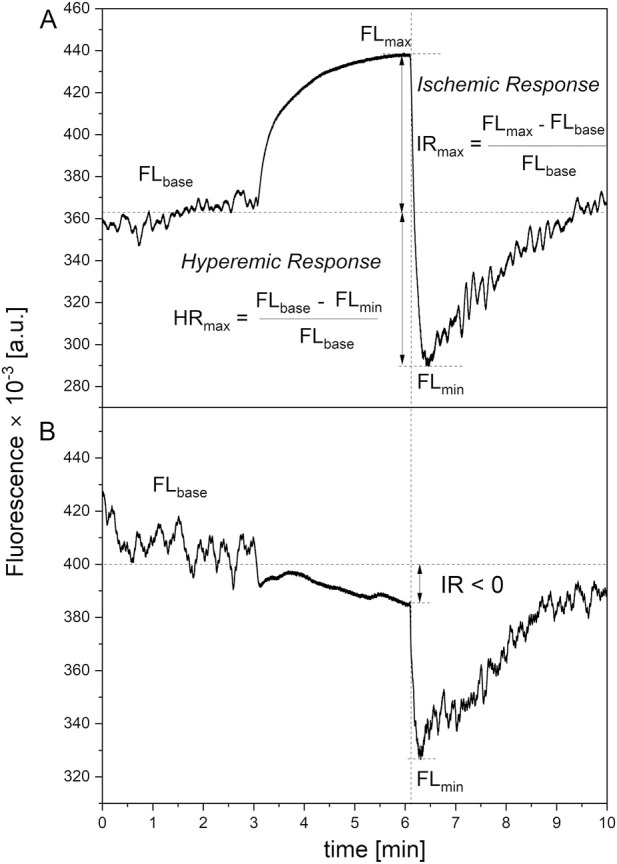
Exemplary FMSF-PORH traces: **(A)** optimal metabolic and vascular function, healthy male, age 44 y.; **(B)** metabolic dysfunction with preserved vascular function, male with obesity, BMI = 35, age 31 y. Abbreviations: FL_base_–the level of NADH fluorescence under normoxia conditions; FL_max_ and FL_min_–maximal and minimal increase/decrease of NADH fluorescence caused by brachial artery occlusion; IR_max_, Ischemic Response maximal; HR_max_, Hyperemic Response maximal; HS, Hypoxia Sensitivity–the strength of oscillations seen after FL_min_ point.

**FIGURE 2:**
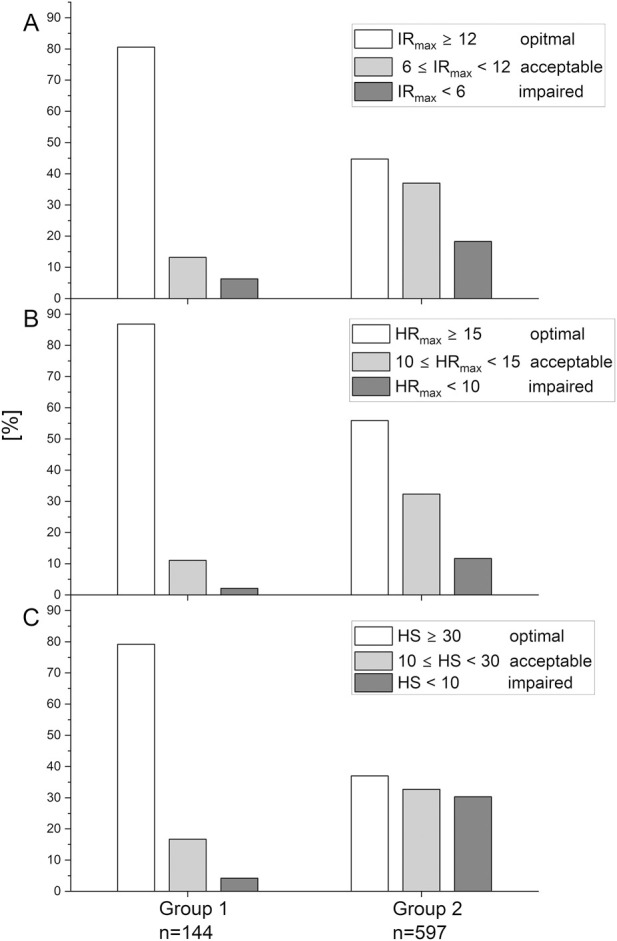
Assignment of individuals (by percentage) to different diagnostic ranges (optimal, acceptable and impaired) of the IR_max_ **(A)**, HR_max_ **(B)** and HS **(C)** parameters in Group 1 (healthy individuals, n = 144, Median age 30 y. (interquartile range IQR: 22–40)) and Group 2 (patients with cardiovascular health problems, n = 597, Median age 68 y. (IQR: 60–75)).

Generally, the FMSF–PORH technique monitors changes in the intensity of NADH fluorescence from the skin on the forearm, which are induced by blocking and releasing blood flow in the brachial artery using an occlusion cuff. An exemplary FMSF trace is presented in Figure 1. The measurement is divided into three phases. The first phase measures NADH fluorescence at the baseline under normoxic conditions. The second phase, which lasts 3 min, measures NADH fluorescence at ischemia. Finally, the hyperemia/reperfusion phase is initiated by the abrupt release of the pressure in the occlusion cuff. Microcirculatory oscillations are visible on the baseline and the reperfusion line, including myogenic oscillations (0.052–0.15 Hz) strongly enhanced upon hypoxia (Marcinek et al., 2023; Marcinek et al., 2024). The three most important parameters characterizing vascular blood flow and metabolic regulation under such conditions will be discussed in the next section. Based on many years of extensive research involving a large number of tested individuals, appropriate reference ranges have been determined for these parameters (Ischemic Response maximal–IR_max_ [%], Hyperemic Response maximal–HR_max_ [%], Hypoxia Sensitivity–HS [a.u.]; see Figure 2 for their optimal, acceptable and impaired ranges). These ranges account for both individual health status and age-related effects (see the difference in the distributions among healthy individuals: Group 1, and older patients with cardiovascular problems; Group 2 in the assigned reference ranges).

### 2.2 The groups studied

The analysis studied two groups: Group 1 – healthy volunteers (77) and amateur and endurance athletes (76); Group 2–910 patients with cardiovascular health problems associated directly with cardiovascular disease (CVD; all: 475, female (f): 273, male (m): 202) or accompanying other diseases, including arterial hypertension (AH; all: 508, f: 285, m: 223), type 2 diabetes (DM2; all: 273, f: 120, m: 153), varicose ulcers (VU; all: 115, f: 52, m: 63), obesity (all: 369, f: 221, m: 148).

The male subgroup among healthy volunteers is significantly larger than the female group because it includes amateur and professional athletes who underwent an endurance test to exhaustion that is not used for women (Bugaj et al., 2019). As no significant differences were observed upon expansion of the male subgroup, the results for the larger group are presented.

The selection of two contrasting groups—young healthy individuals and older patients with cardiovascular health problems—allows for a clear indication of sex-dependent differences that appear with age and accompanying diseases, which are the subject of this study. The characteristics of both groups were described in a previous publication, which was focused mainly on the diagnostic role of myogenic microcirculation oscillations (Marcinek et al., 2025b). Some sex-related differences in vascular circulation and metabolic regulation associated with vascular aging or disease were observed in an earlier investigation (Mikosiński et al., 2023). However, that study included a much smaller group of patients and did not include a healthy group.

### 2.3 Statistical analysis

Statistical analyses were performed using OriginPro 2024. Since the analysed values are not subject to a normal distribution, all of them are characterized by the median and median absolute deviation (MAD) or interquartile range (IQR = Q3–Q1). Comparisons between the two independent groups were analysed using the Mann–Whitney *U* test. The Spearman rank correlation coefficient was used to assess the strength and direction of the correlations between variables. A *p*-value <0.05 was considered statistically significant.

## 3 Results and discussion

### 3.1 Key FMSF-PORH parameters used for the assessment of vascular circulation and metabolic regulation

The NADH/NAD^+^ ratio can be considered a sensitive marker of changes in the functioning of mitochondria and blood vessels, which are responsible for transporting oxygen and other nutrients to cells. Changes in the NADH/NAD^+^ ratio ultimately lead to excessive NADH accumulation (hypoxia) or its reduction (hyperoxia). Any fluctuations (oscillations) in NADH fluorescence reflect variations in the delivery of oxygen to epidermal cells, caused by microvascular oscillations. Therefore, monitoring NADH fluorescence enables the assessment of the NADH/NAD^+^ balance in disorders resulting from dysfunctional vascular circulation and metabolic regulation.

Changes in the absolute level of NADH fluorescence, as measured by the FMSF–PORH method, can be compared individually, indicating the occurrence of reductive stress accompanying various diseases (Mercola, 2025; Yang et al., 2025). The reductive stress caused by strenuous physical exertion can be observed as a strong increase in the skin NADH fluorescence (Bugaj et al., 2019). A remedy for such reductive stress can be the intake of inorganic NO3^−^ or dietary nitrate, which increases nitric oxide (NO) *in vivo* and transiently decreases the level of basal NADH fluorescence (Jurga et al., 2024). The ability of increased NO bioavailability to mitigate the effects of reductive stress have been discussed previously (Marcinek et al., 2025a).

Unfortunately, the absolute level of NADH fluorescence cannot be used for standardized diagnostics of patients, due to individual skin characteristics, differences in skin pigmentation and technical factors related to the measurement of fluorescence. Therefore, it is impossible to establish reference ranges. However, any changes induced by a well-defined external factor can and do provide a diagnostic measure of vascular circulation and metabolic regulation. One such standardized factor forcing the mitochondrial and vascular response is the post-occlusive reactive hyperemia (PORH) method, which involves occlusion of the brachial artery for a sufficient period (e.g., 3 min). While the PORH method is mostly limited to observing changes during hyperemia and reperfusion following the occlusion period, the FMSF–PORH technique also allows for monitoring changes during occlusion, and thus for separate assessment of the vascular and mitochondrial responses due to the complete blockage of blood flow.

The three most important diagnostic parameters of the FMSF–PORH method are related to the response to occlusion. Two of these parameters, characterizing the period of hyperemia and reperfusion, have been described in relation to numerous diseases, particularly cardiovascular diseases and diabetes (Katarzynska et al., 2019; Marcinek et al., 2023; Marcinek et al., 2024; Marcinek et al., 2025a; Marcinek et al., 2025b; Mikosiński et al., 2023).

The most important parameter characterizing the hyperemia period is Hyperemic Response (HR_max_), which is a measure of the maximum decrease in NADH fluorescence after the release of blood flow in the brachial artery (see Figure 1A). This decrease occurs as oxygen is rapidly delivered into the epidermal cells, relative to the resting flow, due to the release of NO and the dilation of the brachial artery. This process can be correlated with the results obtained from Flow Mediated Dilation (FMD) and mainly concerns the macrocirculation.

The most important feature of the reperfusion period (starting at FL_min_ in Figure 1) following the hyperemia stage is the strong activation of microcirculatory oscillations, in particular myogenic oscillations (0.052–0.15 Hz). The intensity of these myogenic oscillations is called Hypoxia Sensitivity (HS). Assessment of the HS parameter that reflects the microcirculatory response to hypoxia allows for diagnosis of microcirculatory dysfunction in diabetes, cardiovascular disease, peripheral arterial disease, and hypertension, as well as the assessment of exercise tolerance (Marcinek et al., 2023; Marcinek et al., 2025b; Marcinek et al., 2025a). An analogical parameter can be provided by Laser Doppler Flowmetry (LDF) measurements (Zhao et al., 2024).

The third important parameter measured by the FMSF-PORH technique is the Ischemic Response (IR_max_) parameter, which corresponds to the maximum change in NADH fluorescence occurring during this stage (usually after 3 min of occlusion, see Figure 1A). This value typically reaches about 15% percent relative to the baseline fluorescence in healthy individuals, but in patients suffering from various diseases it typically reaches much lower values. The kinetics of this process reflects the utilization of residual oxygen after occlusion, as well as the change in the mechanism of cellular respiration itself (Glancy et al., 2021; Pawlak-Chomicka et al., 2023; Marcinek et al., 2025a). Decreases in the IR_max_ parameter may reflect the reductive stress accompanying various diseases. It is worth emphasizing that the same factors which in the short term reduce or increase the level of ischemic response also cause increases or decreases in the level of basal fluorescence, indicating a change in mitochondrial efficiency. This allows us to assign both these effects to the mitochondrial reaction.

FMSF–PORH patterns of the type shown in Figure 1A characterized almost 70% of the entire analysed population of 1063 patients. For further analysis, both groups (1 and 2) were divided into two subgroups with IR_max_ > 0 (144 out of 153 individuals in group 1, and 597 out of 910 in group 2) and IR_max_ ≤ 0 (322 individuals). Only for the FMSF–PORH traces with IR_max_ > 0 can all three parameters (IR_max_, HR_max_, HS) be unambiguously determined. In the case of traces with IR_max_ ≤ 0 only the HS parameter remains unambiguous.

The main results obtained for the group with IR_max_ > 0 are presented in Table 1 and in Figure 2.

**TABLE 1:** Main results obtained for the studied groups.

​	Group 1			Group 2			Group 1 + Group 2 correlations with age			
	Female	Male	*p*	Female	Male	*p*	Female		Male	
							*r*	*p*	*r*	*p*
N	34	110	​	312	285	​	346		395	
Age [years]	30.0 ± 10.5	30.5 ± 8.0	​	69.0 ± 8.0	68.0 ± 7.0	​	​		​	
IR_max_ [%]	16.8 ± 2.2	16.2 ± 4.0	0.92	10.1 ± 4.3	12.2 ± 3.8	**	−0.37	****	−0.42	****
HR_max_ [%]	21.5 ± 2.7	19.8 ± 2.7	0.32	16.8 ± 3.3	14.5 ± 3.2	****	−0.17	**	−0.48	****
HS [a.u.]	86.3 ± 49.8	65.0 ± 39.0	0.53	21.2 ± 15.0	18.3 ± 12.7	*	−0.47	****	−0.48	****

Group 1 – healthy individuals. Group 2 – patients with cardiovascular health problems. Continuous variables, median ± MAD (median absolute deviation); Statistics, *r*–Spearman correlation coefficient, *p*–*p*-value; (*p < 0.05, **p < 0.01, ***p < 0.001, ****p < 0.0001); Mann-Whitney *U* test: female vs. male.

Abbreviations: IR_max_, Ischemic Response maximal; HR_max_, Hyperemic Response maximal; HS, hypoxia sensitivity.

The distribution of the healthy population assigned to the appropriate diagnostic ranges is similar for all three parameters, but in the group with cardiovascular health problems some variation is observable, depending on the parameter examined (Figure 2). In all cases, a deterioration of the results is observed, as evidenced by the decreasing number of optimal results (from around 80%–85% in the healthy population down to around 35%–55% in the older group) in favour of the results assigned to the acceptable and impaired subgroups.

A weak positive correlation (Spearman correlation coefficient, *r* = 0.36; *p*–value <0.0001) was observed between IR_max_ vs. HS (both parameters depend on calcium ion homeostasis) and HR_max_ vs. HS (both parameters depend on NO release during hyperemia and the reperfusion stage). The correlation is almost insignificant between IR_max_ vs. HR_max_ (*r* = 0.19; *p*–value <0.0001), as they relate to completely different vascular circulation statuses (complete flow cessation *versus* increased blood flow during hyperemia).

Among the FMSF-PORH traces, there are also cases where the ischemic response drops below the basal NADH fluorescence value—in some cases even by several percent—almost as a mirror image of the positive signal course (subgroups with IR_max_ ≤ 0). In such cases, we can speak of a negative, abnormal response to occlusion (significantly impaired ischemic response) (see Figure 1B). Although the mechanisms of atypical ischemic responses remain unclear, atypical FMSF–PORH signal patterns may be the result of impaired metabolic processes associated with the loss of or altered mitochondrial functionality. In the group of 910 patients with cardiovascular health problems, almost 40% of women demonstrated such an abnormal response to occlusion, accompanied by a significant reduction in the microvascular response to hypoxia (HS = 8.2 ± 6.3 [a.u.]). In the case of men, only approximately 27% from group 2 demonstrated an abnormal, negative response to occlusion, also with a statistically significant decrease in the HS parameter (HS = 7.2 ± 5.2 [a.u.]). No significant change in the HR_max_ parameter defined as in Figure 1A was observed for negative, abnormal responses to occlusion. However, it should be noted that in the case of waveforms where the occlusion period ends with fluorescence significantly below the baseline value (Figure 1B), a decrease in the NADH fluorescence signal due to increased blood flow after removal of brachial artery occlusion (hyperemic response) cannot clearly be defined.

A small percentage of individuals with impaired vascular circulation can also be found among those who reported no cardiovascular problems, even among professional endurance athletes (Marcinek et al., 2023).

### 3.2 Sex-specific differences in the measured FMSF–PORH parameters characterizing vascular and metabolic health

The high diagnostic potential of the FMSF–PORH technique based on its three key parameters, IR_max_, HR_max_ and HS, allows for precise, non-invasive characterization of vascular and metabolic health. A key challenge for sensitive diagnostic techniques is not only differentiating healthy and sick individuals, but also monitoring subtle effects related to the deterioration of health with age. Tracking vascular and metabolic (mitochondrial) aging is a key issue in preventive medicine. It is generally known that the life expectancy of females is higher than for males, but our understanding of this difference is limited. Therefore, we decided to investigate sex-specific differences in the measured FMSF–PORH parameters characterizing vascular and metabolic health.

As shown in Table 1, all three key FMSF–PORH parameters characterizing vascular circulation (HR_max_ and HS) and metabolic regulation (IR_max_) negatively correlate with age and health problems, both for females and males. Interestingly, the most significant difference between female and men is seen for the HR_max_ parameter, where the decrease of this parameter measured in percentage points (pp) is much slower in females (−0.059 ± 0.016 pp/year) compared to males (−0.128 ± 0.013 pp/year). This difference suggests slower aging of macrovascular function in females, which is generally consistent with the literature (Patel et al., 2005; Skaug et al., 2013; Blum, 2023; Fekete et al., 2025; Freedman et al., 2026).

As shown in Table 1, there are no differences between females and males in the key FMSF–PORH parameters characterizing vascular circulation and metabolic regulation among young healthy individuals (Group 1). Practicing endurance sports at an amateur or professional level does not change this conclusion. Even expanding the analysed group to include male highly trained athletes does not allow for statistically significant differentiation from healthy female volunteers based on FMSF–PORH parameters.

In the older group with cardiovascular health problems (Group 2), the HR_max_ parameter strongly differentiates females and males, indicating better macrovascular function in females, partially because of the slower aging of macrovascular function in females mentioned above. The better macrovascular function seen in females may also be linked to lower arterial stiffness, due to lower vascular wall calcification (Wen et al., 2015; Kim et al., 2021). This also may contribute to the better microvascular hypoxia response (HS) in women.

In contrast, the IR_max_ parameter in the female subgroup from Group 2 is much lower than in the male subgroup, indicating poorer metabolic regulation among females. This is also evidenced by the larger number of abnormal responses to occlusion in women in the group of 910 patients with cardiovascular health problems (almost 40%) compared to men (approximately 27%).

**FIGURE 3:**
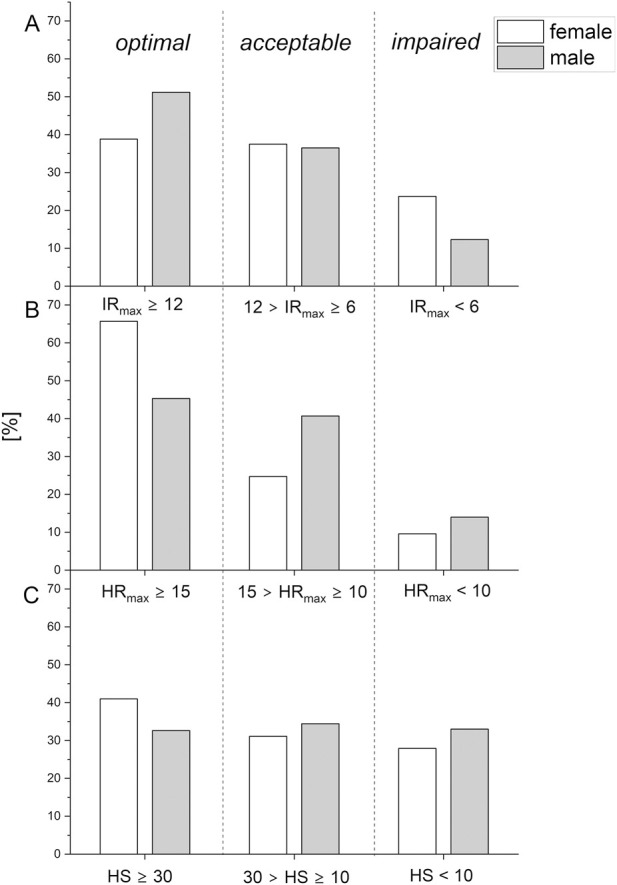
Assignment of individuals (by percentage) to different diagnostic ranges (optimal, acceptable and impaired) of the IR_max_ **(A)**, HR_max_ **(B)** and HS **(C)** parameters in Group 2 divided by sex in subgroups.

These differences are clearly visible when we compare the distributions of individuals from Group 2 in the optimal, acceptable and impaired diagnostic ranges for the FMSF–PORH parameters, in the subgroups characterizing females and males (Figure 3). The general conclusion can be drawn that metabolic dysregulation measured by the IR_max_ parameter is more frequent in females compared to males (IR_max_ < 6 is observed in 24% of females, 12% of males), while females have much better vascular function, as measured by the HR_max_ (HR_max_ ≥ 15 is observed in 66% of females, 45% of males) and HS parameters (HS ≥ 30 is observed in 41% of females, 33% of males).

The observed differences between females and males in terms of vascular and metabolic health are very important and should be taken into consideration in the medical treatment of patients. However, such differences in metabolic (mitochondrial) health status are currently poorly recognized. Very low values for the IR_max_ parameter can be linked to lactate formation, which is regarded as a sensitive marker of metabolic dysregulation in women. For example, elevated blood lactate levels are seen in metabolic dysfunction-associated fatty liver disease in type 2 diabetes patients (Ma et al., 2023; Darshi et al., 2024). On the other hand it is known that calcium overload can affect mitochondrial metabolism and can be directly linked to metabolic health (Walkon et al., 2022). Mitochondrial calcium homeostasis can be strongly mediated by estrogen and such effects may differentiate the metabolic health status of women before and after menopause (Jiao et al., 2020; Koek et al., 2021; Lu et al., 2025). Estrogen has been observed to have a similar effect on vascular circulation (Blum, 2023; Fekete et al., 2025). Our results seem, therefore, to be in accordance with the literature.

The above observations related to sex-specific differences, particularly in metabolic function, require a revision of existing approaches and assumptions, and should initiate clinical research programs devoted to better understanding such fundamental health problems affecting the aging population.

## 4 Conclusion

The FMSF–PORH technique can be applied successfully for the identification of individuals with impaired vascular circulation and/or metabolic (mitochondrial) regulation. Monitoring NADH fluorescence FMSF–PORH enables the assessment of the NADH/NAD^+^ balance in disorders resulting from dysfunctional vascular circulation and metabolic regulation.

Although the sample of 910 patients with cardiovascular disease across diagnostic categories is large enough to draw some conclusions, this group is too small to conduct multivariate analysis controlling for not only heterogeneous diagnoses but also other aspects of health, disease history and severity, treatment modality, *etc.* Due to this limitation, this article does not distinguish between aging and health problems but rather highlights sex-specific differences in the context of these factors.

The following conclusions can be drawn from this study.

- There is no sex-specific difference in the IR_max_, HR_max_ and HS parameters for young healthy individuals (Group 1).
- A statistically significant sex-specific difference was observed in all three parameters for older patients with cardiovascular health problems (Group 2). Females compared to males have a significantly lower IR_max_ parameter, characterizing metabolic (mitochondrial) regulation. In contrast, the HR_max_ parameter characterizing macrocirculation and HS parameter characterizing microcirculation are higher in females compared to males due to lower vascular calcification in females.
- All three parameters, IR_max_, HR_max_ and HS, significantly decrease with age and health problems. However, the decrease of the HR_max_ parameter is much less pronounced in females.

## Data Availability

The original contributions presented in the study are included in the article/supplementary material, further inquiries can be directed to the corresponding authors.
